# The utility of artificial vestibular stimulation in decoding the pathophysiology of mal de débarquement syndrome

**DOI:** 10.3389/fneur.2025.1560787

**Published:** 2025-03-24

**Authors:** Jun Maruta

**Affiliations:** ^1^Department of Neurology, Icahn School of Medicine at Mount Sinai, New York, NY, United States; ^2^Department of Rehabilitation and Human Performance, Icahn School of Medicine at Mount Sinai, New York, NY, United States

**Keywords:** bone conducted vibration, galvanic vestibular stimulation (GVS), irregular vestibular afferents, motion adaptation, nystagmus, spatial orientation, velocity storage, vestibulo-ocular reflex (VOR)

## Mal de débarquement syndrome

Mal de débarquement syndrome (MdDS) is an under-recognized and poorly understood illness. Typically triggered by prolonged exposure to passive motion during a voyage on a cruise ship or airplane, MdDS is primarily characterized by a continuous phantom perception of oscillatory self-motion such as rocking, swaying, or bobbing, or a sensation of gravitational pull (collectively identified as non-spinning vertigo) and associated sensations of imbalance (1–3). The self-motion symptoms of MdDS are generally accompanied by somatic complaints (e.g., headaches and visually induced dizziness), reduced cognitive functions (e.g., decreased attention and short-term memory), and affective problems (e.g., depression and anxiety). These symptoms can be severe enough to often lead to long-term disability (4–6) and for some patients to develop suicidal thoughts (7, 8).

Transient mal de débarquement (commonly known as “sea legs”), representing the common after-sensation that mimics the exposed physical motion and associated postural instability, has been recognized for centuries (9, 10). Although MdDS, a chronic manifestation of mal de débarquement, has gained increasing recognition following the 1987 publication of a six-patient case series (1), the illness has yet to permeate the awareness of clinicians and is often misdiagnosed as a mental disorder, vestibular migraine, or dizziness caused by peripheral vestibular dysfunction. Patients are said to typically make 2–5 visits to healthcare professionals before their MdDS diagnosis, but those who undergo 20 or more such visits are not uncommon (2, 4, 5, 11). As such, the actual prevalence of the illness cannot be determined presently, but at least a small percentage of patients seen at large clinical centers specializing in balance and dizziness are identified as having MdDS (12, 13) and ~80% of reported cases are women (4, 6, 14, 15).

The pathogenesis of MdDS is poorly understood, but the experience of mal de débarquement suggests some form of motion-induced entrainment in the central vestibular pathways (16–19). MdDS is not associated with an injury or overt structural change in the peripheral or central nervous system, but is rather thought to be a disorder generated from synaptic changes that can be reversed (14, 19–22). Most people spontaneously recover from transient mal de débarquement within hours to several days (16, 21). Why the condition persists into a chronic form in some people, or how accompanying symptoms develop is not clear.

The likelihood of spontaneous remission is said to decrease with time (2, 23). Further, once diagnosed with MdDS, patients face limited treatment options. Although MdDS is believed to hinge on central vestibular processing, conventional vestibular physical therapy generally offers little benefit (6, 19, 24). Some patients experience partial symptom relief from benzodiazepines, a class of GABA-A agonists (6, 19, 21), but the site of their pharmacological action is not understood, and harmful effects including dependence must be considered (25, 26). Other patients may experience improved quality of life with vestibular migraine medications, but symptom improvement appears domain-specific, and patients' sensitivity to medications may require a greater degree of dose management than typical (27, 28).

It was against this backdrop that a possible link was discovered between MdDS and the velocity storage mechanism of the central vestibular system (20, 29). This discovery opened opportunities for positive long-term outcomes of MdDS through approaches that target the neural malleability of velocity storage, thereby addressing the root cause of the illness (14, 20, 30–32).

## Velocity storage

**Figure 1:**
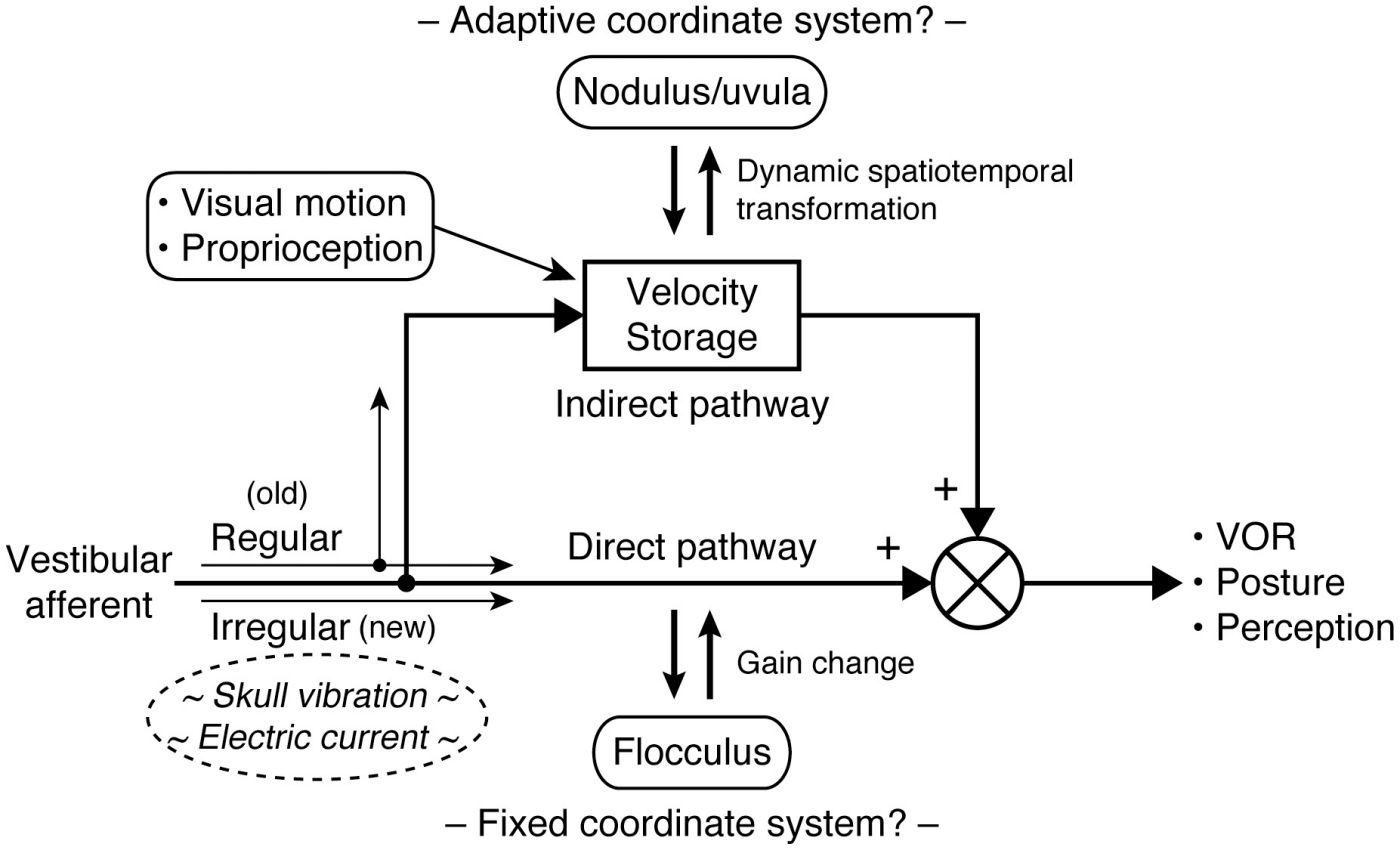
Central vestibular processing conceptualized with parallel direct and indirect pathways. In the VOR, the direct pathway corresponds to the three-neuron arc of the reflex and interacts with the cerebellar flocculus to calibrate its output. The indirect (velocity storage) pathway involves multiple sensory integration and interacts with the cerebellar nodulus and uvula to support a unified sense of self-motion and stability. Type I and type II hair cells in the vestibular organs are respectively synapsed by calyceal and bouton endings of afferent fibers, which in turn are associated with irregular and regular patterns of signaling. Phylogenetically, velocity storage and type II vestibular hair cells antecede type I vestibular hair cells. As it is hypothesized that irregular fibers bypass velocity storage, the difference in the responsiveness of regular and irregular fibers to vibration or galvanic stimuli may be utilized to elucidate pathological vestibular conditions, including MdDS.

Velocity storage is closely associated with the vestibulo-ocular reflex (VOR) as it was first examined as a stored eye movement drive that generally prolongs the vestibular and optokinetic responses beyond the input activity through slow charging and discharging (33–36), but it can also be activated by proprioceptive cues for continuous rotation (37–39). Indeed, velocity storage is a center of multimodal sensory integration. In addition to ocular reflexes, velocity storage is thought to contribute to postural control (31, 40) and the perception of self-motion (34, 35, 37, 41, 42). Ocular, postural, and perceptual responses are often conceptualized as the sum of the outputs of the velocity storage and non-velocity storage pathways, with the latter directly relaying peripheral sensory activity (Figure 1) (31, 33–35, 40, 42). Sectioning vestibular commissural fibers caudal to the abducens nucleus selectively abolishes the sluggish VOR components attributed to velocity storage while sparing fast direct responses, supporting the presence of the separate neural pathways (43, 44).

Velocity storage does not merely prolong the signals received from peripheral sensors, but actively reconstructs and dynamically reshapes information about self-motion, embodying a more dynamic working-memory like quality than short-term memory (45). For example, off-vertical axis rotation (OVAR) in darkness induces continuous compensatory nystagmus by activating the otolith organs, which are not rotation sensors in a normal sense (46). Similarly, stepping in place on a circular treadmill in darkness induces nystagmus that compensates for the apparent circling (38, 39). Further, per-rotatory nystagmus in response to off-center rotation while facing in or away from the direction of motion develops an out-of-plane, vertical component as the centripetal acceleration tilts the gravito-inertial field sideways (47). Critically, in order for velocity storage to interpret the incoming information and perform coordinate transformations in these manners, it needs to maintain its own referential representation of three-dimensional space.

Studies of the ocular and postural reflexes have revealed that sensorimotor transformation in the brain may be facilitated by the use of a common coordinate frame consistent with the orthogonal arrangement of the semicircular canals (48–52). Such transformation is aided by external cues related to the constant presence of gravity in the terrestrial environment (46, 53–56). However, this arrangement is established through motion exposure-dependent neuroplasticity and can undergo changes in an unusual acceleration environment (29, 52, 55, 57, 58). Damage to the cerebellar caudal vermis (nodulus and uvula) compromises the ability to generate nystagmus during OVAR or reorient eye velocity to the gravito-inertial field (59–62). Therefore, the coordinate transformations in the indirect (velocity storage) pathway are shaped through brainstem-cerebellar interactions and are also subject to the flexibility of the internal reference frame. In contrast, the direct pathway appears to operate on a fixed coordinate system whose outcome is determined by gain calibration and vector summation of separate channels (Figure 1) (63–65).

MdDS is thought to result from a failure in velocity storage to readapt to a normal acceleration environment after adapting to passive motion (29, 58). A treatment approach designed to correct the presumably maladapted spatial properties of velocity storage with a combination of visual and vestibular stimuli has significantly improved the overall outcomes of MdDS, with a success rate of ~80% and ~50% in short and long terms, respectively (14, 15, 20, 30–32, 66–70). Still, substantially many patients do not benefit from the treatment, and the neural basis of the illness is far from clear.

## Transient symptom improvement by passive motion

Physical signs associated with MdDS such as postural imbalance and direction-changing nystagmus are not present in all patients nor unique to MdDS, nor do they indicate the subjective severity of the illness (3, 20). Instead, the diagnosis of MdDS is based solely on clinical history and subjective reports (3, 71). The symptoms of MdDS greatly overlap with those of another chronic vestibular disorder known as persistent postural perceptual dizziness (PPPD) (72), which with MdDS may represent a large spectrum of non-spinning vertigo (3, 6). However, exposure to passive motion typically worsens symptoms of PPPD while temporarily alleviates those of MdDS (2, 3, 71). Thus, the effect of passive motion may be a critical difference between MdDS and PPPD (3).

The effect of passive motion also reveals the ability of inner-ear-driven signals to modulate symptoms of MdDS. The mechanism for this phenomenon is not known. However, in experimental animals, some neurons in the vestibulo-olivo-cerebellar pathway of the caudal vermis were found with oscillatory entrainment in their spontaneous activity after exposure to minutes of cyclic tilting motion, but this oscillatory activity was transiently reset by, rather than superimpose with, vestibular signals consisting of other frequencies, only to resume when the stimulus stopped (73). The direct relevance of this finding to MdDS is not clear, but caudal vermal representation of adaptation to a moving environment as well as transient cessation of such activity by new motion exposure seem significant given the area's close relevance to velocity storage.

## Artificial vestibular stimulation

Galvanic vestibular stimulation (GVS) affords stimulation of vestibular primary afferents without moving the head, by instead passing low electrical current through the skin over the mastoids. GVS is becoming widely applied to both experimental and clinical testing of balance functions in health and neurological conditions (74, 75), but not yet in MdDS. GVS is also used to generate head motion cues during interactions in virtual/simulated environments (76–78).

Cathodal and anodal current respectively activates and silences vestibular afferent fibers, with lower threshold for irregular than regular afferents (74, 75, 79–81). By vector summation, directionally different responses can be elicited. For example, bilateral bipolar stimulation with the anode on the right side induces a rightward postural sway (or an overall illusory leftward rotational perception in immobilized subjects), and bilateral monopolar anodal stimulation with a distant reference electrode induces a backward postural response (74, 77, 78). Under certain conditions, blindfolded human subjects can be steered by remotely-controlled GVS while walking (82). Bilateral bipolar injection of noise current increases the activity of afferent fibers in a stochastic manner (75) to induce a non-specific self-motion perception in normal subjects, reported as “weird” but generally more comfortable and less irritating or nauseating than GVS with square-wave pulses of equivalent current amplitude (83).

Bone conducted vibration (BCV) applied to the skull at ~100 Hz is another means to stimulate vestibular primary afferents without moving the head, other than the vibration motion itself, typically with an amplitude of 1 mm or less at an intensity of a body massager (84–86). BCV reportedly highly selectively activates irregular afferents (87–89), but is otherwise a global vestibular stimulus because skull vibration stimulates afferent nerves from both ears. Normally, the effects of bilaterally activated vestibular nerves are said to be negated centrally because of the push-pull organization of the vestibular system; however, unilateral dysfunction creates a response asymmetry, forming the basis of the skull vibration-induced nystagmus (SVIN) test (84, 86). The safety and sensitivity of the SVIN test are well established (84, 85).

SVIN in individuals with unilateral vestibular loss appears and disappears abruptly at the onset and cessation of BCV, respectively, without a progressive buildup in intensity or slow-decaying after-nystagmus (90, 91). This characteristic is different from the charging and discharging behavior of velocity storage with a time constant of at least several seconds even with unilateral vestibular loss (92, 93). Further, interference by BCV to the nystagmic response to cold caloric stimulation to the intact ear is similarly abrupt and in accordance with the amplitude and duration of SVIN, indicating separately sustained velocity storage activity during BCV (91). Therefore, a possible “tilt dump” effect (94) from the concurrent stimulation of otolithic afferents during BCV may be ruled out as an explanation for the lack of after-nystagmus associated with SVIN.

From such unique characteristics and mechanism underlying SVIN, it is hypothesized that irregular afferents bypass the velocity storage mechanism (Figure 1) (95, 96). By contrast, regular afferents' contribution to velocity storage is supported by the existing knowledge. Vestibular type I and type II hair cells, respectively contact calyceal and bouton endings of afferent fibers, which are roughly associated with irregular and regular firing patterns (96, 97). Vestibular type I hair cells are phylogenetically new, as anamniotes (fishes and amphibians) only have type II vestibular hair cells, but some such species demonstrate well-functioning velocity storage (98–101). Further, the VOR during sinusoidal rotation or OVAR in genetically manipulated mice with deficient type I hair cell development does not suggest impaired velocity storage (97). A note of possible significance, however, is that GVS and BCV may have different selectivity for irregular afferents, as modest after-nystagmus has been observed to accompany GVS-induced nystagmus (102, 103). Indeed, the intensity of GVS, thus the level of recruitment of more regular afferent fibers, that is required to induce eye movement is known to be higher than that for inducing postural or perceptual responses (74, 80, 81, 104).

Although the neurological etiology and factors that contribute to the development of MdDS are not clear, several mechanisms have been postulated, including maladaptation of velocity storage (20, 29), entrainment in the cerebral networks (105), vestibular migraine (27, 28), and hormonal dysregulation (106, 107). Since MdDS is not associated with peripheral damage, the SVIN test may at first glance seem to have little to inform the pathophysiology of the illness. However, it is not yet known if patients with MdDS will experience temporary alleviation of their symptoms with BCV or low-intensity GVS. As these stimuli selectively or preferentially activate irregular afferents, a negative result would indicate a specific role of regular afferents in modulating symptoms of MdDS during passive motion as well as indirectly support the velocity storage-basis of MdDS pathogenesis. A positive result may indicate a different pathogenic mechanism and necessitate a revision to the current understanding of MdDS, but would also indicate the potential utility of these stimuli in the diagnosis or treatment of the illness. The velocity storage mechanism may still be accessed with high-intensity GVS, which may find its own utility. Lastly, premorbid asymmetry in the vestibular sensitivity may contribute to the activation of velocity storage while naturally interacting in the environment, possibly making the individual more susceptible to MdDS during exposure to passive motion (58). BCV or GVS may also be useful in probing such a possibility.

## Conclusion

MdDS is a debilitating vestibular disorder whose pathophysiology is presently little understood. An animal-based model of MdDS would be useful but has not been established yet (29, 58, 108). BCV and GVS are well-studied artificial vestibular stimuli that may be safely tested on patients. Since these stimuli selectively or preferentially activate irregular vestibular afferents and may, perhaps to a different degree, bypass the velocity storage mechanism, studying their effects may fill a piece of the puzzle of MdDS pathophysiology.
